# Cell cycle-specific phosphorylation of p53 protein in A549 cells exposed to cisplatin and standardized air pollutants

**DOI:** 10.3389/fphys.2023.1238150

**Published:** 2023-08-14

**Authors:** Agata Niechoda, Katarzyna Milewska, Joanna Roslan, Karolina Ejsmont, Adam Holownia

**Affiliations:** Department of Pharmacology, Medical University of Bialystok, Bialystok, Poland

**Keywords:** A549, alveolar epithelial cells, cisplatin, DNA damage, nanoparticle carbon black, p53 phosphorylation, urban dust

## Abstract

Exposure to particulate matter is associated with DNA damage and the risk of lung cancer. Protein p53 is activated by multi-site phosphorylation in the early stages of DNA damage and affects cell outcome. Our study aimed to assess the effect of (100 µg/mL^−1^/24 h) standardized air pollutants: carbon black (CB), urban dust (UD), and nanoparticle carbon black (NPCB) on cell cycle, DNA damage and 53 phosphorylation at Ser 9, Ser 20, Ser 46, and Ser 392 in proliferating and quiescent A549 cells and in cells that survived cisplatin (CisPT) exposure. Phosphorylated p53 was quantified in cell subpopulations by flow cytometry using specific fluorochrome-tagged monoclonal antibodies and analysis of bivariate fluorescence distribution scatterplots. CisPT, UD and NPCB increased site-specific p53 phosphorylation producing unique patterns. NPCB activated all sites irrespectively on the cell cycle, while the UD was more selective. p53 Ser 9-P and p53 Ser 20-P positively correlated with the numbers of CisPT-treated cells at G0/G1, and NPCB and NPCB + CisPT produced a similar effect. A positive correlation and integrated response were also found between Ser 20-P and Ser 392-P in resting A549 cells treated with NPCB and CisPT but not UD. Interdependence between the expression of p53 phosphorylated at Ser 20, and Ser 392 and cell cycle arrest show that posttranslational alterations are related to functional activation. Our data suggest that p53 protein phosphorylation in response to specific DNA damage is driven by multiple independent and integrated pathways to produce functional activation critical in cancer prevention and treatment.

## 1 Introduction

Exposure to particulate matter (PM) is associated with an increased risk of lung cancer (Hamra et al., 2014). Clinical and experimental data show that PM induces lipid peroxidation, protein oxidation, and DNA damage (Møller et al., 2014; Niu et al., 2020). PM nanoparticles (NPs), molecules between 1 and 100 nm diameter can directly induce oxidative stress by interaction with the cellular redox system and generation of intracellular reactive oxygen species (ROS) or indirectly, via inflammation and cytokine release (Li et al., 2010). Oxidative damage of DNA by ROS and reactive nitrogen species (RNS) is a widely accepted mechanism of NP-induced genotoxicity (Lopes et al., 2017; Pandey and Prajapati, 2018). NP produce both structural and functional modifications of DNA, induce DNA damage, activation of p53, and mobilization of proteins related to DNA repair (Shukla et al., 2021). DNA damage responses are regulated by chromatin structure, but histones are also targets for NPs. Hammond et al. (2003) reported that p53 Ser 15 and histone H2A.X were both phosphorylated in response to hypoxia by ataxia telangiectasia (ATX) and Rad3-related protein kinase. We have previously shown that short exposure to the model particle, i.e., urban dust (UD) caused both single- (SSB) and double-strand DNA breaks (DSB) in alveolar adenocarcinoma-derived epithelial non-small cell lung cancer (A549) cells (Mroz et al., 2008). A549 cells however are phenotypically distinct. Some subpopulations of A549 cells respond to a model DNA-damaging drug–cisplatin (CisPT) with cycle arrest, “early” G0/G1 cell accumulation, and apoptosis, while others are resistant to CisPT (Xu et al., 2016; Horibe et al., 2022). Both kinds of cell types differ in DNA damage response. DNA double-strand breaks are repaired by non-homologous end joining, which re-ligates the broken ends of the DNA and homologous recombination (Li J. et al., 2019). P53-binding protein 1 (53BP1) plays a crucial role in non-homologous repair (Eliezer et al., 2014; Li L. et al., 2019). In the CisPT-resistant fraction of A549 cells, there is increased expression of murine double minute 2 (MDM2) which plays an important role in G2/M arrest and apoptosis (Han et al., 2019). Also, significantly higher ATM and p53 protein was detected in A549 cells treated with CisPT (Eliezer et al., 2014). Although the genome guardian role of p53 has mainly been studied regarding apoptosis, many findings suggest that p53 is activated also in the early stages of DNA damage. Hammond et al. (2003) reported that p53 Ser 15 and histone H2A.X were both phosphorylated in response to hypoxia and reoxygenation-induced DNA damage. Distinct phosphorylation sites in p53 may have highly specific roles in cell biochemistry but functional synergism between the two or more sites should be considered (Zhu X. et al., 2023; Shen et al., 2023). On the other hand some phosphorylation sites may play only regulatory roles. Phosphorylation of p53 at C-terminal Ser392 was reported to increase DNA-specific binding while N-terminal Ser 15, 19, and 20 may be crutial crucial for the release of p53 from its negative regulator MDM2 (Wu et al., 2019). Our study aimed to assess the effect of coarse carbon black (CB), standardized urban dust (UD), and nanoparticle carbon black (NPCB) on p53 activation in proliferating and quiescent alveolar epithelial cells (A549 cell line) and cells that survived CisPT exposure and to associate, if possible, distinct phosphorylation sites of p53 with functional cell cycle regulation.

## 2 Materials and methods

### 2.1 Reagents

All chemicals used in the present study were obtained from Sigma Chemical (Poznan, Poland) unless otherwise stated, while cell culture media and reagents were obtained from GIBCO (Thermo Fisher Scientific, Waltham, United States).

### 2.2 Cell culture

A549 cells obtained from American Type Culture Collection (ATCC; Manassas, United States), were grown in Ham’s F-12K Nutrient Mixture (Sigma Chem. Co., Poznan, Poland) supplemented with 10% fetal bovine serum, 100 U/mL penicillin, 100 μg/mL streptomycin, and 2 mM L-glutamine (GIBCO/BRL; Grand Island, United States) in Falcon flasks (Fisher, Poznan, Poland) at 37.5°C in an atmosphere of 95% air and 5% CO2. Cells expressing wildtype p53 protein were grown as monolayers in asynchronous phases by trypsinization and reseeded before reaching subconfluency. After the fifth passage, the cells were allowed to attach, quiesced overnight in serum-free media, and subsequently treated under serum-free conditions.

A549 cells were also pretreated overnight with cisplatin (CisPT; 30 μg mL^−1^) which was then removed and the culture medium was replaced with a PM-conditioned medium for 24 h.

### 2.3 Particles and treatment

Culture media supplemented with PMs were prepared using commercial, standardized urban dust (UD; Standard Reference Material 1649b, the particle size 0.2–110 nm, with a mean size of about 10 nm), which was purchased from the National Institute of Standards and Technology (Gaithersburg, United States), nanoparticle carbon (NPCB; 14 nm diameter, Printex 90; Degussa, Frankfurt, Germany), while coarse carbon black (CB; 260 nm diameter, Huber 990; Haeffner and Co. Ltd., Chepstow, United Kingdom) was used as a reference substance. The particles were suspended in a serum-free culture medium at a concentration of 100 µg/mL^−1^ and were sonicated in a Bandelin Sonoplus ultrasonic homogenizer for the 30 s before use. A549 cells were quiesced overnight in serum-free media and were subsequently treated with PMs for 24 h.

### 2.4 Cytotoxicity and cell cycle

Determination of cell viability and proliferation was estimated by flow cytometric quantification of the cellular DNA, using propidium iodide (PI) staining in permeabilized cells. Briefly, cellular DNA degradation and cell cycle analysis were performed on cells stained for 30 min with PI (50 mg mL^−1^) in Tris buffer (100 mM, pH 7.5) containing potassium cyanide (0.1%; w/v), Nonidet-P40 (0.01%; w/v), RNase III-A (40 mg mL^−1^, 4 KU mL^−1^) and NaN_3_ (0.1%; w/v). The analysis was performed on an aligned Beckman Coulter CytoFlex flow cytometer (Beckman Coulter, Warsaw, Poland). PI fluorescence was measured in ≥5,000 cells with appropriate bandpass filters. DNA histograms were further analyzed by DNA quantification software (Kaluza, V2.1.2, Brea, CA, United States). Cells were quantified by their relative distribution in the damaged-hypodiploid phase (“early” G0/G1 zone of the DNA fluorescence histograms), diploid phase (G0/G1 zone, pre-DNA synthesis/resting), S-phase (DNA synthesis) and G2/M phase (post-DNA synthesis/mitosis).

### 2.5 Expression of phosphorylated p53 proteins

**FIGURE 1:**
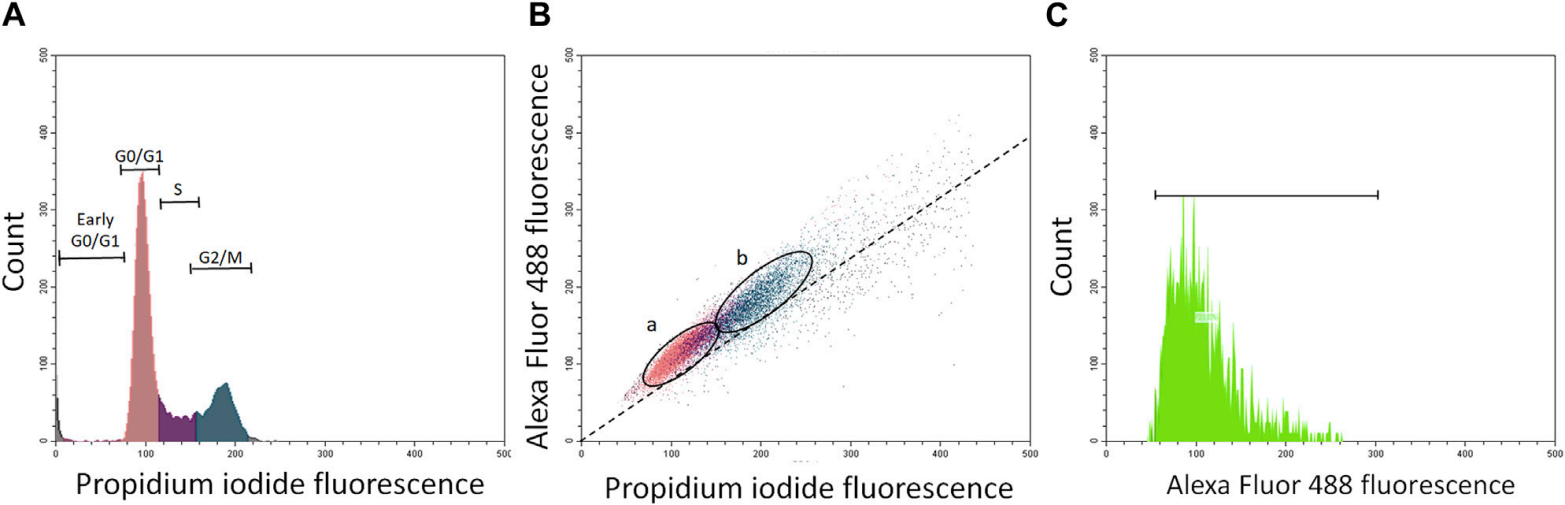
Panels **(A–C)** show flow cytometry quantification of phosphorylated p53 protein in about 5000 resting and proliferating A549 cells (ratio about 2:1). Panel **(A)**—histogram of propidium iodide (PI)-stained cells with damaged cells (gated as early G0/G1 cells), resting cells gated as G0/G1 cells (sienna), S phase cells (violet), and G2/M cell (ocean). Panel **(B)**-bivariate scatterplots reflecting cellular DNA content (red fluorescence from propidium iodide) vis a vis green (Alexa Fluor 488) stained phosphorylated p53 in resting cells (G0/G1; **(A)** and proliferating cells (S + G2/M; **(B)**. Cells were gated by “paint-a-gate” and mean (green) fluorescence from phosphorylated p53 was quantified in **(A)** and **(B)** fractions as shown on the scatterplot **(B)** and histogram **(C)**. The dashed skewed line **(B)** represents the lower threshold level.

Phosphorylated p53 was analyzed by flow cytometry using specific monoclonal antibodies and positive and negative controls (Figures 1A–C). Cells were fixed for 15 min in 2% buffered formalin, washed twice in PBS and once with 0.1% Triton X-100 (Sigma Chemical Co., Poznan, Poland) in PBS followed by a 1% solution of bovine serum albumin (BSA) in PBS for 30 min to block nonspecific binding. The cells were then incubated in 100 μL volume of 1% BSA containing 1:100 dilution of phosphospecific (Ser 9, Ser 20, Ser 46, or Ser 392) mouse monoclonal antibodies (Cell Signaling). After overnight incubation at 4°C, the cells were washed twice with PBS and then incubated with the fluorochrome-tagged secondary antibody (100 μL of 1:100 dilution of AlexaFluor488 from Invitrogen/Molecular Probes, Eugene, OR) for 45 min at room temperature in the dark. Cell fluorescence was measured on a Beckman Coulter CytoFlex flow cytometer (Beckman Coulter, Warsaw, Poland) and shown as bivariate distribution scatterplots (Figure 1B).

### 2.6 Statistical analysis

To analyze whether phosphoprotein expression was increased, the significance of a difference between treated and untreated cells was assessed using a one-sample *t*-test or a one-way ANOVA with Bonferroni’s multiple comparisons test as appropriate. Differences were considered to be statistically significant at *p*-values <0.05. Linear regressions and correlations between cell numbers in resting and proliferating fractions of A549 cells and site-specific phosphorylated p53 levels and between different specific phosphorylated p53 were calculated according to the Pearson test.

## 3 Results

**FIGURE 2:**
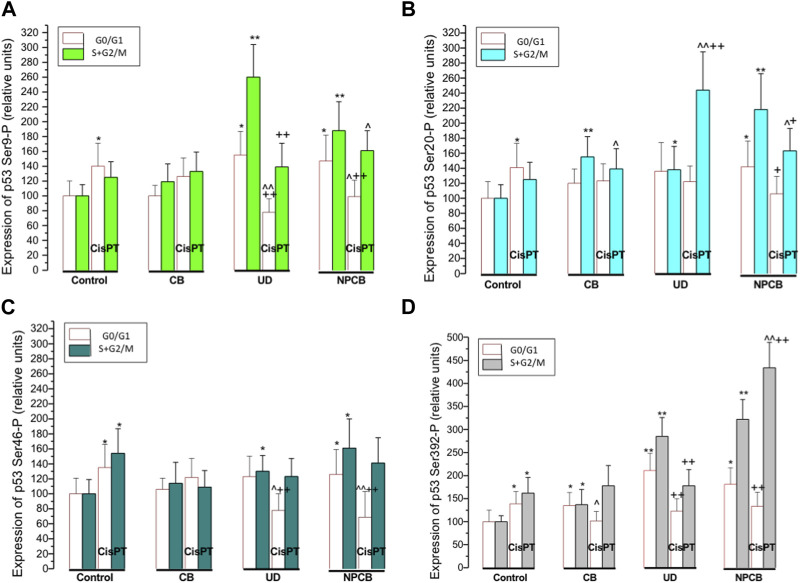
Panels **(A–D)** show bars representing relative expression of p53 protein phosphorylated at Ser 9 (panel A) Ser 20 (panel B), Ser 46 (panel C) and Ser 392 (panel D) in naïve A549 and A549 cells pretreated with cisplatin (CisPT; 30 μg mL^−1^) and then treated for 24 h with coarse carbon black (CB), standardized urban dust (UD; Standard Reference Material 1649b) or nanoparticle carbon (NPCB). CB was used as a reference substance. The particles were suspended in a serum-free culture medium at a concentration of 100 μg mL^−1^. The phosphorylated p53 protein was quantified by flow cytometry in about 5000 cells using specific monoclonal antibodies and bivariate scatterplots reflecting cellular DNA content (red fluorescence from propidium iodide) vis a vis green (Alexa Fluor 488) from phosphorylated p53 in resting cells in G0/G1 phase and proliferating cells in S + G2/M phase.

Table 1 and Figures 2A–D show the effect of carbon black (CB), urban dust (UD), and nanoparticle carbon black (NPCB) on cycle-specific phosphorylation of p53 protein at Ser 9, Ser 20, Ser 46, and Ser 392 in subfractions of nonproliferating (G0/G1) and proliferating (S + G2/M) naïve A549 cells and cells pretreated with CisPT (CisPT).

**TABLE 1:** The effect of carbon black (CB), urban dust (UD), and nanoparticle carbon black (NPCB) on cycle-specific phosphorylation of p53 protein at Ser 9, Ser 20, Ser 46, and Ser 392 in subfractions of nonproliferating (G0/G1) and proliferating (S+G2/M) naïve A549 cells and cells pretreated with CisPT (CisPT). Specific phosphoproteins were quantified by flow cytometry using specific monoclonal fluorescent antibodies. Cell fluorescence was measured in 5000 cells on a Beckman Coulter CytoFlex flow cytometer and shown as bivariate distribution scatterplots and histograms.

	Control	CisPT	CB	CB + CisPT	UD	UD + CisPT	NPCB	NPCB + CisPT
p53 Ser 9-P								
G0/G1	100 ± 20	140 ± 31*	110 ± 14	126 ± 25	155 ± 32*	78 ± 18^^++	147 ± 35*	99 ± 22^++
G2/M	100 ± 15	125 ± 21	119 ± 24	133 ± 26	260 ± 44**	139 ± 32++	188 ± 39**	161 ± 27^
p53 Ser 20-P								
G0/G1	100 ± 22	141 ± 32*	120 ± 19	123 ± 23	136 ± 28	122 ± 21	142 ± 34*	106 ± 23+
G2/M	100 ± 18	125 ± 23	155 ± 27**	139 ± 27	138 ± 31*	244 ± 51^^++	218 ± 48**	163 ± 30^+
p53 Ser 46-P								
G0/G1	100 ± 21	135 ± 31	106 ± 15	122 ± 25	123 ± 27	78 ± 22^++	126 ± 33*	69 ± 34^^++
G2/M	100 ± 19	154 ± 33**	114 ± 28	109 ± 22^^	130 ± 21*	123 ± 24	161 ± 39*	141 ± 34
p53 Ser 392-P								
G0/G1	100 ± 25	139 ± 26*	135 ± 28*	102 ± 20^	211 ± 37**	123 ± 27++	181 ± 36*	133 ± 3++
G2/M	100 ± 13	162 ± 34**	137 ± 33*	178 ± 44	285 ± 41**	178 ± 35++	322 ± 43**	434 ± 55^^++

*P<0.05; ** P<0.01 for comparisons with control cells.

^ P<0.05; ^ ^ P<0.01 for comparisons with CisPT-treated cells.

+P<0.05; ++P<0.01 for comparisons with corresponding UD or NPCB-treated cells.

CisPT increased expression of phosphorylated Ser 9 (*p* < 0.05) and Ser 20 (*p* < 0.05) in the N-terminal transactivation domain and Ser 392 (*p* < 0.05) in C-terminal regulatory domain in resting cells (G0/G1 phase) which survived CisPT pretreatment. In proliferating cells (S + G2/M) CisPT increased phosphorylated p53 at Ser 46 (*p* < 0.01), and Ser 392 (*p* < 0.01). Slight increases at Ser 9-P and Ser 20-P in S + G2/M were not significant.

CB elevated Ser 392-P (*p* < 0.05) in resting and proliferating cells and increased Ser 20-P (*p* < 0.01) only in S + G2/M cells. CB normalized Ser 46-P (*p* < 0.05) elevated by CisPT in cells at the G2/M phase and decreased (*p* < 0.05) p53 Ser 392-P in a regulatory domain at G0/G1 phase.

UD increased activation of all sites at G2/M and Ser 9-P (*p* < 0.05) and Ser 392-P (*p* < 0.01) at the G0/G1 phase of the cell cycle. Surprisingly, Ser 9-P was significantly lower (*p* < 0.01) in resting (G0/G1) cells treated with UD + CisPT than in cells treated with CisPT only, while the level of Ser 20-P in the same group but at G2/M phase was significantly (*p* < 0.01) higher.

NPCB resulted in the activation of all sites, irrespectively on the phase of the cell cycle. In CisPT pretreated cells NPCB produced a decrease of Ser 9-P (*p* < 0.05) and Ser 46-P (*p* < 0.01) in the G0/G1 phase and an increase in G2/M Ser 9-P (*p* < 0.05), and Ser 20-P (*p* < 0.05), and a very high increase in Ser 392 (about 3 folds; (*p* < 0.01).

Regression and correlation between the expression of p53 phosphorylated at Ser 9, Ser 20, Ser 46, Ser 392 and G0/G1 or S + G2/M cell numbers were performed in selected groups with elevated phosphorylated p53. In cells treated with CisPT p53 Ser 9-P and Ser 20-P positively correlated (*r* = 0.82; *p* < 0.01 and *r* = 0.65; *p* < 0.05, respectively) with the numbers of CisPT “resistant” cells at GO/G1 phase of the cycle. A similar effect in G0/G1 cells was observed in Ser 20-P and Ser 392-P in NPCB and NPCB + CisPT, where corresponding r values were 0.77 (*p* < 0.05) and 0.66 (*p* < 0.05). It should be noted that regressions and correlations between G2/M cell numbers and activated p53 were usually negative, but not significant.

A positive correlation was also found in G0/G1 cells treated with NPCB between Ser 20-P and Ser 392-P (*r* = 0.67; *p* < 0.05).

## 4 Discussion

Exposure to low-size PM increases the incidence of lung cancer (Badyda et al., 2016; Consonni et al., 2018; Zhu XZ. et al., 2023). ^32^P-labelling experiments have shown that PM increases the probability of mutations and cancer (Vineis and Husgafvel-Pursiainen, 2005). Other experimental studies, including ours, also evidenced that both PM and volatile pollutants are mutagenic (Mroz et al., 2008; Pan et al., 2023). CisPT is a model compound for experimental DNA damage. The drug usually stops the cell cycle in G0/G1 phase (Miyata et al., 2015). CisPT is widely used in therapies for different types of cancer including non-small cell lung cancer (NSCLC). The initial efficiency is usually high, but most tumors gradually become drug-resistant (Galluzzi et al., 2012; Kryczka et al., 2021). The role of p53 in chemosensitivity and chemoresistance remains unclear, but increased transcriptional activity of 53 may be important in reversing CisPT resistance (Hernandez-Valencia et al., 2018). CisPT resistance is common in A549 cells which are derived from NSCLC. DNA damage by CisPT activates the p53 tumor suppressor protein which acts as a transcription factor that regulates the expression of genes involved in cell survival or apoptosis and blocks the proliferation of damaged cells (Sui et al., 2022). It has been reported that acquisition of CisPT resistance occurs in cancer cells expressing wild-type p53 (Cao et al., 2020). The p53 protein is regulated by transcription, translation, turnover, compartmentalization, and regulatory proteins, but even naïve A549 cells show the presence of the constitutively expressed p53 phosphorylated at Ser 9, Ser 20, Ser 46, and Ser 392 in interphase and mitotic nuclei. Our study aimed to assess the effect of CB, UD, and NPCB on DNA damage and p53 multisite phosphorylation in proliferating and quiescent A549 cells and in cells that survived CisPT exposure, assuming that their response to noxious PM stimuli is different. The regulation of multi-site phosphorylation is extremely complex, but it seems, that even small changes in p53 activity can affect cell outcomes. The p53 protein has several phosphorylation sites at the N-terminal transactivation domain, a central DNA-binding domain, an oligomerization domain, and a C-terminal regulatory domain (MacLaine and Hupp, 2011). We have previously found, that PM increases the phosphorylation of p53 at Ser 15, which is required for activation and apoptosis but not for p53 stabilization (Mroz et al., 2004). p53 is usually activated in the early stages of DNA damage-promoting signaling events, which leads to the repair of damaged cells (Lee et al., 2010). The main functional event in the induction of p53 protein is the uncoupling of p53 from degradation, mediated by murine double minute 2 (MDM2) protein (Kulikov et al., 2010; Haupt et al., 1997). MDM2 reduce significantly p53 response (Shi and Gu, 2012; Meek and Hupp, 2010), and this mechanism may play a role in decreased expression of p53 Ser 9-P and Ser 46-P in the G0/G1 phase after UD + CisPT or NPCB + CisPT.

Induction and activation of p53 in response to DNA damage are mediated by the ataxia telangiectasia mutated (ATM), and ataxia telangiectasia and Rad3-related protein (ATR) protein kinases, which are activated by double- and single-strand breaks respectively (de Lange, 2018). Our results show that G0/G1 cell cycle arrest may be related to overexpression of p53 at Ser 9-P and at Ser 20-P, which were highly elevated after CisPT and/or NPCB exposure. Cell arrest at the G1 phase by p53 may serve for the repair of potentially lethal DSB, and increase survival of damaged cells. In our early paper functional activation of p53 via single phosphorylation was studied in lung cancer patients on chemotherapy (Mroz et al., 2004). Increased phosphorylated p53 at Ser 20 was found in patients on radiotherapy/CisPT/vinorelbine and correlated with proliferation marker Ki67 and elevated poly (ADP-ribose) levels (Mroz et al., 2004). We have also shown that increased phosphorylation of 53 at Ser 15 tracks UD and NPCB but not CB exposure (Mroz et al., 2008). Now, increased p53 phosphorylation at Ser 9 Ser 20, Ser 46 and Ser 392 was found in cells which resisted CisPT. The drug usually stops the cell cycle in G0/G1 phase. Our data show that G0/G1 cells have elevated phosphorylated p53 mostly in the transactivation zone (at Ser 9 and Ser 20). G2/M cells express higher levels of p53 phosphorylated at Ser 46 and the C-terminal regulatory domain, while G2/M cells express mostly p53 Ser 46-P. It shows that the p53 phosphorylation profile changes with the cell cycle. Ser 46, an N-terminal phosphorylation site has discriminatory functions for p53 as a transcriptional activator (Li J. et al., 2019). Phosphorylation of Ser 46 is induced by relatively severe DNA damage and is selectively pro-apoptotic (Yoshida et al., 2006).

C-terminal Ser 392 of p53 is constitutively phosphorylated in unstressed cells but is increased by genotoxic agents (Castrogiovanni et al., 2018). We have shown a positive correlation between the expression of p53 Ser 20-P and p53 Ser 392-P in G0/G1 cells treated with NPCB. It is possible, that this response is integrated and has a role in the functional activation of the p53 protein. Interdependence between the expression of p53 phosphorylated at Ser 20, and Ser 392 and cell cycle arrest show that this posttranslational alteration may be related to functional activation especially after DNA damage by CisPT or NPCB but not UD.

Our experimental data are consistent with the role of p53 in modulating the activation of cell cycle checkpoints, especially at highly conserved Ser 20 and Ser 392 sites relevant to transactivation and stabilization, respectively (Fraser et al., 2010). It is possible, that individual phosphorylation sites in p53 have specific roles, but there is an apparent synergism between specific sites. Taken together, the DNA damage activates specific patterns of p53 protein phosphorylation, and identification of their functional patterns may be critical in cancer prevention and treatment.

## Data Availability

The raw data supporting the conclusion of this article will be made available by the authors, without undue reservation.
